# How to avoid genu recurvatum in leg-length discrepancy treated with tension-band plates. A volumetric magnetic resonance analysis

**DOI:** 10.1016/j.jor.2024.06.004

**Published:** 2024-06-08

**Authors:** Maria Jurado-Ruiz, Luis Riera, César G. Fontecha

**Affiliations:** a Department of Orthopedic Surgery and Traumatology, Vall d'Hebron University Hospital, Universitat Autònoma de Barcelona, Passeig de la Vall d'Hebron, 119, 08035, Barcelona, Spain; b Reconstructive Surgery of the Locomotor System, Vall d'Hebron Research Institute, Universitat Autònoma de Barcelona, Passeig de la Vall d'Hebron, 129, 08035, Barcelona, Spain; c Department of Pediatric Radiology, Vall d’Hebron University Hospital, Passeig de la Vall d'Hebron, 119, 08035, Barcelona, Spain; d Department of Pediatric Orthopedics and Traumatology, Sant Joan de Déu Hospital, Passeig de Sant Joan de Déu, 2, 08950, Esplugues de Llobregat, Barcelona, Spain

**Keywords:** Temporary epiphysiodesis, Growth plate, Guided growth, Leg length discrepancy, Tension-band plates, Genu recurvatum, Magnetic resonance analysis

## Abstract

**Aims and objectives:**

Genu recurvatum deformity after treatment of leg-length discrepancy (LLD) with tension-band plating is a recognized, but poorly described phenomenon in medical literature. The aim of this study was to evaluate clinical and radiological features of patients treated with tension-band plating for LLD assessing the development of a recurvatum deformity and its relationship to plate and screw disposition in a transversal plane, thus attempting to establish optimal plate positioning.

**Materials and methods:**

Retrospective study of children with LLD treated with tension-band plating. Primary endpoints were clinical and radiological knee recurvatum and anterior and posterior physeal areas measured drawing a line spanning from the lateral to the medial tension-band plates in the transverse plane using volumetric magnetic resonance imaging (vMRI). These findings were compared between patients with and without knee recurvatum.

**Results:**

Twelve children (mean age 11.7 years) were included. Average follow-up was 2.6 years (1.5–5.0). Tension-band plating led to a significant reduction in LLD (mean, 15 mm). Six patients (50 %) developed clinical genu recurvatum (mean, 22°). According to vMRI, patients with genu recurvatum had a larger posterior to anterior physeal area ratio in both distal femur (1.6 versus 0.9, *p* < 0.05) and proximal tibial physes (2.2 versus 1.0, *p* < 0.05).

**Conclusion:**

The optimal position of the tension-band plates in distal femoral and proximal tibial physes should be in a point where a posterior to anterior physeal areas ratio is around 1.0, so as to achieve an even distribution of the physeal areas in the multidimensional physeal transverse plane. This point anatomically corresponds in the sagittal X-ray view to an imaginary line located just anterior to the posterior diaphyseal cortical bone on a true lateral radiograph for both femur and tibia.

## 1 Introduction

Leg-length discrepancy (LLD) is a frequent reason for referral to paediatric orthopaedists.^1^ This problem may be treated by temporary epiphysiodesis with tension-band plates or percutaneous transphyseal screws implanted on both sides of the physeal growth plate in the femur and/or tibia of the longer extremity. The aim is to halt or relent growth and allow the shorter limb to reach the same length as the longer one.^2^ The use of this procedure has increased in recent years, mainly because gradual deformity correction not only avoids the need for more extensive surgery (e.g., osteotomy), but it is also reversible, allowing for growth resumption once the implant is removed.^3^ However, there are concerns in the literature questioning the effectiveness of tension-band plating in LLD and in regard to the risk of developing secondary deformities such as genu varus, genu valgus and genu recurvatum.

Genu recurvatum is a knee deformity in which the tibiofemoral joint has an extension range of motion beyond 20°,^4^ causing altered gait biomechanics.^5^ Clinically, the deformity is characterized by knee hyperextension with a posterior pseudo drawer sign and a sensation of weakness and instability of the knee. It is associated with impaired proprioception during extension and an increased risk of anterior cruciate ligament rupture,^6^ posterior knee pain, and residual instability.^7^ Bone remodelling does not occur in adulthood, thus predisposing patients to early osteoarthritis through compression of the medial femorotibial joint leading to anteromedial pain.^7^^,^^8^

Few articles mention genu recurvatum as a complication of temporary epiphysiodesis in the treatment of LLD in children, suggesting that the deformity could occur as a result of excessively anterior placement of plates^9^, ^10^, ^11^ or percutaneous transphyseal screws.^12^ A centred physeal position of the implants might prevent a later sagittal deformity.

The aim of this study was to analyse whether an asymmetric distribution of anterior and posterior physeal areas on volumetric magnetic resonance imaging (vMRI) measurements is associated with a recurvatum deformity and whether their symmetrical distribution does not produce this disorder. Therefore, we also wanted to determine the proper implant location on the distal femoral and proximal tibial epiphyses in order to prevent secondary genu recurvatum. To our knowledge, this is the first study to use vMRI to explore possible causes of genu recurvatum after tension-band plating.

## 2 Materials and methods

### 2.1 Study design and population

We performed a retrospective review of our institutional database to identify all patients with LLD treated with guided growth using tension-band plating from January 1, 2013 to December 31, 2017 and who had a minimum follow-up of 1 year. We selected those patients who developed a postoperative genu recurvatum and we compared them with a matched group who did not develop this deformity. The study was approved by our institution's ethics committee (CEIC) (Number PR(AMI)321/2017).

### 2.2 Outcome variables

The main outcome determinations were clinical genu recurvatum, radiological recurvatum, and vMRI physeal measurements. Demographics, diagnosis, date of surgery, and preoperative and final LLD were also recorded.

All patients were evaluated clinically by an independent observer who measured recurvatum deformity in degrees using a standard goniometer.

### 2.3 Radiographic assessment

Standard radiographic protocol obtained preoperatively and at follow-up included a standing teleoroentgenogram of the legs, where lower limb length was measured from the upper end of the femoral head to the middle of the distal tibial articular surface. Conventional lateral radiographic views of both knees were obtained routinely to measure and to compare the posterior distal femoral angle (PDFA) and the posterior proximal tibial angle (PPTA) preoperatively and postoperatively. The normal value for PDFA is 83° ±3° and the normal value for PPTA is 81° ± 3°. PDFA and PPTA assessment in children was made modifying the method described by D. Paley,^13^ as we were using conventional lateral X-Ray views due to technical impossibility to obtain routinely standing lateral views in full extension.

### 2.4 MRI technique and data analysis

**Fig. 1:**
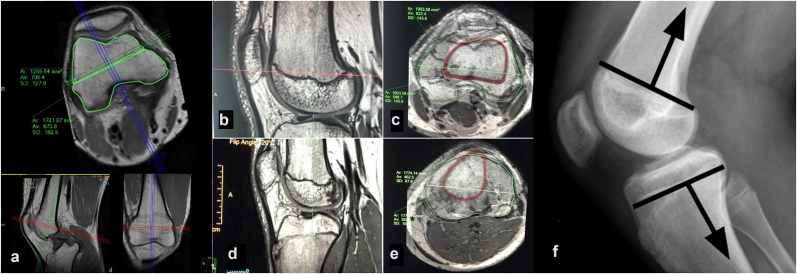
**vMRI measurements in a distal femoral physis of a child**. We can observe the axial plane at the level of the distal femoral physis of the knee (a). We then calculate the anterior and posterior physeal areas relative to the line that crosses the physis from both medial to lateral tension-band plates holes. In this case, there is a mild uneven distribution of anterior and posterior femoral physeal areas (posterior to anterior area ratio of 1.4). Images b-e show vMRI measurements in a normal, non-intervened knee, so as to find the location of a line that distributes anterior and posterior areas equally. Distal femoral physis in sagittal (b) and axial (c) views. Volumetric magnetic resonance measures in proximal tibial physis in sagittal (d) and axial (e) views. In both axial views we overlap the physeal area (green outline) to the diaphyseal area (red outline); this allows us to translate the optimal position of tension-band plates on true lateral radiographs (e). We suggest that that the line that distributes equally anterior and posterior areas is just anteriorly to the posterior cortex in both femur and tibia.

An MRI analysis was performed in every patient at the end of follow-up, by using volumetric acquisition SPACE (Sampling Perfection with Application optimized Contrasts using different flip angle Evolutions, Siemens, Enlargen Germany), which consists of a single-slab 3D TSE sequence with slab-selective, variable excitation pulse and proton density–weighted imaging in the sagittal plane. Volumetric acquisition allows multiplanar reconstructions with minimal susceptibility artefacts in an acceptable time frame and without signal-to-noise ratio limitations.^14^^,^^15^ Using advanced multiplanar reconstruction software, the axial planes of the knee were oriented to calculate the anterior and posterior physeal areas relative to a line that crosses the physeal area from the center of both the medial to the lateral tension-band plates at the distal femur and proximal tibia (Fig. 1a). Physeal areas were measured in mm^2^.

After obtention and process of vMRI images, we overlapped the axial plane image at the level of the physeal section to the axial plane image at the level of the diaphyseal section (Fig. 1, b-f). We hypothesized this method could allow us to correlate the line that distributes anterior and posterior physeal areas equally, to a point in the lateral X-ray view. We believe that knowing the position of this point with respect to the anterior and posterior femoral and tibial corticals would help us more accurately locate the optimal position of the TBP, thus drawing a longitudinal and parallel line that crosses this point.

### 2.5 Tension-band plating operative technique

The senior author (C.G.F.) performed all cases. The surgery is described open and percutaneous. In our review, all the cases were performed through open surgery, following the surgical technique described by Stevens et al..^16^, ^17^, ^18^ In the frontal plane, the plate must be centred over the physis so that the screw holes are divergently oriented towards the epiphysis and metaphysis symmetrically. In the lateral view, the plate must be perpendicular to the physis and parallel to the internal aspect of the posterior cortical surface.

### 2.6 Statistical analysis

Quantitative variables were compared using the nonparametric Mann-Whitney U tests for independent data in order to identify statistically significant differences between surgically treated and untreated limbs and between patients with and without genu recurvatum at the end of follow-up. A *p*-value of <0.05 was considered statistically significant.

## 3 Results

### 3.1 Baseline characteristics

We reviewed our clinical database and located a total of 27 patients who had undergone tension-band plate epiphysiodesis for LLD at our hospital. However, because of lack of follow-up, only 12 patients (4 females and 8 males, mean age 11.7 ± 3.1 years) were eligible for analysis; these 12 were included in our study. The mean follow-up was 2.6 years. Almost half of the LLD cases (42 %) were idiopathic. Guided growth correction by tension-band plating led to a clinically and statistically significant reduction in mean clinical LLD from baseline (26.5 ± 6.8 mm) to the end of follow-up (11.8 ± 9.9 mm) (p = 0.01) (Table 1). Six of the 12 patients (50 %) developed genu recurvatum in the surgically treated limb; they had all undergone surgery before 2016. Groups with and without knee recurvatum deformity were comparable for age (p = 0.49), sex (p = 0.54) and initial LLD (p = 1.00).

**Table 1:** Baseline characteristics of patients.

Characteristics	Number of patients (N = 12)
Gender (Female/Male)	4/8
Age at surgery ± SD (years)	11.7 ± 3.4
Diagnosis, n (%)	
Idiopathic	5 (41.7)
Longitudinal deficiency	3 (25.0)
Undetermined dysplasia	1 (8.3)
Hemihypertrophy	1 (8.3)
Juvenile idiopathic arthritis	1 (8.3)
Perthes	1 (8.3)
Recurvatum, n (%)	
Yes	6 (50.0)
No	6 (50.0)
Discrepancy (SD), mm	
Initial	26.5 ± 6.8
Final	11.8 ± 9.9
p-value	0.01

### 3.2 Radiographic measurements for surgically treated and untreated limbs in patients with and without genu recurvatum

In the group of patients who developed genu recurvatum, mean postoperative PDFA in the surgically treated limb averaged 92.9 ± 9.5° and mean postoperative PPTA was 93.5 ± 6.9° (Table 2). Patients who did not develop recurvatum deformity had a mean postoperative PDFA of 84.6 ± 4.4° and a mean postoperative PPTA of 81.9 ± 6.5° in the surgically treated limb.

**Table 2:** Comparison of radiological measures in patients with clinical recurvatum.

	Recurvatum	No Recurvatum	*p*-value
**PDFA (*±SD)***	92.9 (9.5)	84.6 (4.4)	0.065
**PPTA (*±SD)***	93.5 (6.9)	81.9 (6.5)	0.082

### 3.3 vMRI measurements in the surgically treated knee in patients with and without genu recurvatum

**Fig. 2:**
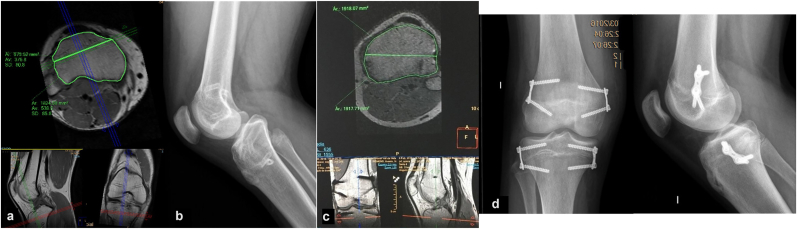
**Comparison of knee vMRI in children with and without clinical recurvatum**. We can observe an uneven distribution of anterior and posterior tibial physeal areas according to the placement of tension-band plates in the axial plane of a proximal tibial vMRI (a) (posterior to anterior physeal areas ratio of 2.7). The patient presented a secondary 15° tibial slope (b) with a clinically significant genu recurvatum. We can observe an even distribution of anterior and posterior tibial physeal areas according to the placement of tension-band plates in the axial plane of a proximal tibial vMRI (c) (posterior to anterior areas ratio of 1.00). The patient did not developed a clinically significant genu recurvatum (d).

The mean anterior physeal femoral area in patients who developed genu recurvatum was 1212.4 ± 284.3 mm^2^, while the mean posterior physeal femoral area was 1830.7 ± 647.1 mm^2^. The mean posterior to anterior area ratio was 1.60 ± 0.62. The mean anterior physeal tibial area was 990.9 ± 360.8 mm^2^ and the corresponding mean posterior physeal tibial area was 1877.6 ± 412.0 mm^2^. The mean posterior to anterior area ratio in tibia was 2.2 ± 1.2 (Fig. 2a and b).

In patients without genu recurvatum, the mean femoral area from the physeal plate was 1506.7 ± 174.1 mm^2^ anteriorly and 1303.5 ± 446.5 mm^2^ posteriorly. The mean ratio between the posterior and anterior femoral areas was 0.9 ± 0.2. The tibial area measured 1523.2 ± 324.9 mm^2^ anteriorly and 1553.8 mm^2^ ± 511.6 mm^2^ posteriorly. The mean ratio between posterior to anterior tibial areas was 1.0 ± 0.3(Table 3).

**Table 3:** vMRI parametrics in patients with and without recurvatum.

		Recurvatum	No Recurvatum	*p*-value
**Distal Femoral Physis**	**Anterior Area (mm**^**2**^**)**	1212.4	1506.7	0.065
	**Posterior Area (mm**^**2**^**)**	1830.7	1303.5	0.132
	**Ratio**	1.6	0.9	0.041
**Proximal Tibial Physis**	**Anterior Area (mm**^**2**^**)**990.9	990.9	1523.2	0.082
	**Posterior Area (mm**^**2**^**)**	1877.6	1553.8	0.429
	**Ratio**	2.2	1.0	0.017

## 4 Discussion

Proper positioning of eight-plates in guided growth for the treatment of LLD is of utmost importance. Secondary coronal and sagittal deformities are described to a varying degree, and both bidimensional malalignments of the knee are known risk factors for the development of osteoarthritis. In this series of patients with LLD treated with tension-band plating, we observed statistically significant differences between patients with and without genu recurvatum deformity in terms of antero-posterior positioning of the plates. As we could objectify through vMRI measurements at the level of both physes of the knee, patients with genu recurvatum had statistically significant larger posterior to anterior physeal areas ratios in both distal femoral physis (1.6 versus 0.9, p < 0.05) and proximal tibial physis (2.2 versus 1.0, p < 0.05) with respect to the sagittal position of the eight-plates.

We also observed differences between patients with and without recurvatum deformity concerning postoperative PDFA and PPTA measurements. Patients with recurvatum tended to have a larger postoperative PDFA in the surgically treated limb than those without clinical recurvatum, although this difference was not statistically significant (p = 0.065). Patients with recurvatum tended to have a larger postoperative PPTA in the operated limb than those without clinical recurvatum, although this difference was also not statistically significant (p = 0.082). This could be due to the small size of our sample, which would have reduced the power of the statistical analysis, or perhaps due to differences in local anatomy between the femur and the tibia.

Althought the relationship between major PDFA and/or major PPTA and the occurrence of recurvatum may be complex and influenced by other factors, such as ligamentous laxity, muscle strength, individual anatomy, and underlying pathological conditions, either or both of these increased parameters may be a contributing factor to recurvatum. In all cases, we consider that a full clinical evaluation and consideration of multiple factors are necessary to fully understand the occurrence of recurvatum in patients treated with TBP due to LLD, as well as to determine appropriate management in individual cases.

Consistent with our results, Furuhashi et al.^19^ suggested that extension deformity of the distal femur occurred frequently after temporary epiphysiodesis and that the deformity was more prominent with anteriorly placed implants. In our series, 6 out of 12 patients developed a genu recurvatum deformity after tension-band plate epiphysiodesis, suggesting that this complication might be underdiagnosed in both clinical practice and research, even taking into account the present study's undeniable limitations.

There are several described surgical techniques to correct sagittal plane knee deformities. Amongst them, anterior distal femoral epiphysiodesis is a surgical technique performed for the treatment of fixed knee flexion deformity in patients with several disorders, including arthrogryposis, neurological and neuromuscular diseases..^20^, ^21^, ^22^ Guided growth for tibial recurvatum has been described by Stevens et al.^23^; in a retrospective review of 5 physes, they report the technique and preliminary results of placing a single extraphyseal TBP in the posterior proximal tibial physis. It is therefore reasonable to imagine that asymmetrical tension-band plate placement in the sagittal plane may cause a similar effect, albeit unfavourable in our scenario.

Based on the obtained results, we can assume that inadequate positioning of the TBP in the sagittal plane can lead to secondary deformities and influence treatment outcomes. According to the radiological results and confirmation by vMRI, we could hypothesize that the optimal femoral and tibial tension-band plate placement site is along a straight line drawn parallel to the internal aspect of the posterior cortical surface on a conventional true lateral projection radiograph. Notwithstanding, optimal plate placement entails some technical difficulties and is not free of intraoperative complications. Posterior positioning of the plate in the distal femur is anatomically simple enough. However, such positioning may put the posterior cortical surface at risk of being perforated or damaged by drills or screws, thus caution during this step is of vital importance. In regard to the proximal tibia, proper posterior placement of the plates is hindered by adjacent anatomical structures: the *pes anserinus* on the medial tibial level, and the peroneal head laterally. In addition, physeal distribution at the proximal tibia is difficult to achieve because the tibia is an axially shaped bone with a triangular prism, resulting in a larger posterior area where its wider base is located.

The clinical course of genu recurvatum is not entirely predictable. It is believed that remodelling may occur in paediatric patients after hardware removal given the persistence of physical adaptability to the new loading requirements. However, genu recurvatum leads to biomechanical alterations in the short term that could affect the development of joint and capsule-ligamentous structures. Given that most children undergo LLD treatment near the end of skeletal maturity, there is little chance for remodelling and, since there is no remodelling in adulthood, persistence of genu recurvatum could lead to anteromedial knee pain and predispose patients to early onset knee osteoarthritis. Furthermore, it is well established in the literature that genu recurvatum increases the complexity of joint replacement in knees with osteoarthritis^24^ and is a predictor of poorer outcome.^25^

Overall, results must be interpreted with caution, as we recognize the limitations of our study. Firstly, the main limitation of our study is the small number of patients analysed. Secondly, this was a retrospective analysis, with all the limitations inherent to such studies, the inability to obtain all relevant data, as well as the necessity of relying upon chart notes. Finally, our study did not follow all patients up to skeletal maturity. As strengths, mention the strong statistical correlation of some of the results obtained and their consistency with other findings in the literature. To our knowledge, this is the first study to measure and compare clinical, radiological, and MRI findings between patients with and without recurvatum deformity after treatment of LLD using tension-band plates.

## 5 Conclusions

As the use of tension-band plating to treat LLD in paediatric patients is becoming increasingly common, genu recurvatum is an iatrogenic complication that may probably be underdiagnosed in both the literature and clinical practice. It occurs following inadequate tension-band plates placement in the sagittal plane of the knee and can be avoided by using intraoperative true lateral radiograph images to fix the plates more posteriorly.

According to our results, the ideal location for tension-band plate placement in the tibia or femur is the point with a posterior to anterior area ratio of 1:1, so as to achieve an even distribution of the physeal areas in the multidimensional transverse plane. This point anatomically corresponds in the sagittal X-ray view to an imaginary line located just before the posterior cortical bone on the lateral radiograph for both femur and tibia.

## Use of AI tool

No use of AI tool.

## Data availability

Available in a repository upon request.

## Patient consent

No patient consent needed due to retrospective nature and public database.

## Ethical approval

All procedures performed were in accordance with the ethical standards of the institutional and/or national research committee and with the 1964 Helsinki declaration and its later amendments or comparable ethical standards. This article does not contain any studies with animals performed by any of the authors. This study was approved by the Ethics Committee of the authors’ institution (approval number: PR(AMI)321/2017).

## Funding

This research did not receive any specific grant from funding agencies in the public, commercial, or not-for-profit sectors.

## Intellectual property

We confirm that we have given due consideration to the protection of intellectual property associated with this work and that there are no impediments to publication, including the timing of publication, with respect to intellectual property. In so doing we confirm that we have followed the regulations of our institutions concerning intellectual property.

## CRediT authorship contribution statement

**Maria Jurado-Ruiz:** Conceptualization, Data curation, Formal analysis, Investigation, Methodology, Project administration, Resources, Software, Visualization, Roles, Writing – original draft, Writing – review & editing. **Luis Riera:** Investigation, Methodology, Project administration, Resources, Software, Supervision, Validation, Visualization, Writing – review & editing. **César G. Fontecha:** Conceptualization, Data curation, Formal analysis, Investigation, Methodology, Project administration, Resources, Software, Supervision, Validation, Visualization, Roles, Writing – original draft, Writing – review & editing.

## Declaration of competing interest

No conflict of interest exists.
